# Virtual-Lattice Based Intrusion Detection Algorithm over Actuator-Assisted Underwater Wireless Sensor Networks

**DOI:** 10.3390/s17051168

**Published:** 2017-05-20

**Authors:** Jing Yan, Xiaolei Li, Xiaoyuan Luo, Xinping Guan

**Affiliations:** 1 Institute of Electrical Engineering, Yanshan University, Qinhuangdao 066004, China; li.xiaolei1989@foxmail.com (X.L.); xyluo@ysu.edu.cn (X.L.); 2 Department of Automation, Shanghai Jiao Tong University, Shanghai 200240, China; xpguan@sjtu.edu.cn

**Keywords:** intrusion detection, sensor, underwater, coverage

## Abstract

Due to the lack of a physical line of defense, intrusion detection becomes one of the key issues in applications of underwater wireless sensor networks (UWSNs), especially when the confidentiality has prime importance. However, the resource-constrained property of UWSNs such as sparse deployment and energy constraint makes intrusion detection a challenging issue. This paper considers a virtual-lattice-based approach to the intrusion detection problem in UWSNs. Different from most existing works, the UWSNs consist of two kinds of nodes, i.e., sensor nodes (SNs), which cannot move autonomously, and actuator nodes (ANs), which can move autonomously according to the performance requirement. With the cooperation of SNs and ANs, the intruder detection probability is defined. Then, a virtual lattice-based monitor (VLM) algorithm is proposed to detect the intruder. In order to reduce the redundancy of communication links and improve detection probability, an optimal and coordinative lattice-based monitor patrolling (OCLMP) algorithm is further provided for UWSNs, wherein an equal price search strategy is given for ANs to find the shortest patrolling path. Under VLM and OCLMP algorithms, the detection probabilities are calculated, while the topology connectivity can be guaranteed. Finally, simulation results are presented to show that the proposed method in this paper can improve the detection accuracy and save the energy consumption compared with the conventional methods.

## 1. Introduction

In recent years, underwater wireless sensor networks (UWSNs) have been proposed to explore the ocean and support solutions for time-critical aquatic applications, such as port surveillance, environment monitoring, disaster prevention and mine reconnaissance; see [1,2,3] and the references therein. Due to the lack of a physical line of defense, intrusion detection becomes one of the key issues in applications of UWSNs, especially when confidentiality has prime importance. For instance, in order to ensure the tracking performance, target surveillance applications often require detecting the absence or presence of an underwater intruder [4].

For terrestrial wireless sensor networks, the intrusion detection problem has been extensively studied, and barrier coverage is one of the most popular strategies to detect the existence of an intruder [5]. Since barrier coverage only needs a few nodes to construct a continuous barrier spanning the protected region, it is particularly efficient and low cost for intruder detection applications compared with area coverage. Early research in barrier coverage is focused on static sensors [6,7,8]. More recent research has examined barrier coverage with mobile sensor nodes. For instance, [9] jointly exploited sensor mobility and intruder arrival information to improve barrier coverage. In [10], a strong barrier coverage problem was investigated where the maximum moving distance of mobile sensor nodes is minimized. In [11], a maximum lifetime barrier coverage problem was investigated, whose goal is to maximize the lifetime of the barrier coverage offered by the hybrid sensor networks. However, these barrier coverage-based algorithms are only suitable for two-dimensional terrestrial environment, and they cannot be directly applied to the three-dimensional underwater environment.

To make up the shortcoming mentioned above, [12] derived the notion of three-dimensional stealth distance to measure how far a submarine can travel in a sensor network before being detected. In [13], a deployment strategy for the three-dimensional underwater environment was proposed, whose objective is to determine the minimum number of sensors to achieve optimal sensing and communication coverage. The work in [14] focused on the connectivity and *k*-coverage issues in the three-dimensional environment, where the minimum sensor spatial density was given to ensure *k*-coverage of sensor networks. To maximize the lifetime of sensor nodes and ensure the quality of intrusion detection, [15] proposed an outermost shell coverage algorithm to guarantee the recognition quality of intruding events in the three-dimensional environment. In these literature works, the nodes are static, and this design limits the autonomy of intrusion detection. For instance, in order to cover an area, the sensor nodes are required to be deployed with perfect positions; however, the static property of sensors and the dynamic property of the current prevent the perfect deployment. Inspired by this, some scholars put forward the deployment of underwater autonomous underwater vehicles (AUVs) to improve the autonomy. For instance, [16] developed the “Suave” (i.e., swarm underwater autonomous vehicle localization) algorithm to localize swarms of AUVs operating in rough waters, whose purpose is to ensure that all AUVs arrive at their destinations by preserving localization throughout the entire mission. Nevertheless, these underwater mobile nodes are very expensive due to the more complex underwater transceivers and to the hardware protection needed in the extreme underwater environment. Then, how to achieve the intrusion detection with a small number of mobile nodes becomes a new issue. Moreover, the transmission power for underwater acoustic communications is much higher than that in terrestrial radio communications, while batteries installed on underwater sensors are harder to recharge. Under these constraints, sensors near the sink that are consistently retransmitting data easily deplete their energy. Based on this, how to save the underwater communication energy becomes another issue. Normally, the so-called neighbor rule is widely used to illustrate the topology relationship of sensor nodes. For instance, a neighbor rule-based routing algorithm was analyzed for underwater static and moving nodes in [17]. Analyzing the network topology with the neighbor rule reveals that many interactions between sensors are redundant. The redundancy sometimes makes the communication complex and inefficient. In our previous work [18,19], a rigid graph-based dynamic coverage strategy was proposed to save the communication consumption of nodes. However, it is still unknown whether the rigid graph-based optimization can improve the intrusion detection performance in the underwater environment.

In this paper, we investigate the intrusion detection problem in the three-dimensional underwater environment, where connectivity and detection probability are both considered. The UWSNs are composed of a large number of SNs and multiple mobile actuator nodes (ANs). The roles of SNs and ANs are to detect the environment and perform monitor patrolling actions based on the detected data, respectively. Compared with traditional static sensor networks, this architecture not only has the capacity of detecting the environment, but also can make decisions based on the observations and perform monitor patrolling actions. On the other hand, only a small number of mobile ANs is used, and then, it is more economical compared with mobile sensor networks. Then, we propose a virtual-lattice-based monitor (VLM) algorithm to detect the intruder. An optimal and coordinative lattice-based monitor patrolling (OCLMP) algorithm is also provided for UWSNs to reduce the redundancy of communication links and improve detection probability. Under the VLM and OCLMP algorithms, the detection probabilities are both calculated, while the topology connectivity can be guaranteed. Comparing with [9], the proposed algorithm does not need to know the intruder prior information. In addition, the energy efficiency in this paper can be improved by compared with neighbor rule-based method [20].

The remainder of this paper is organized as follows: Section 2 includes the problem formulation and preliminaries. The main results of the intrusion detection algorithms are presented in Section 3. To verify the validity of the algorithm, some simulation results are given in Section 4. Finally, the conclusion and future work are given in Section 5.

## 2. Problem Formulation and Preliminaries

In this section, we first give the sensing model of SNs and ANs, then the energy model of the communication consumption is constructed. In addition, the average intruder detection probability is defined, and then, some preliminaries on graph theory are provided. In order to clarify and simplify the algorithms, it is assumed that nodes are time-synchronized and that each node knows its location, from some time synchronization techniques [21] and location schemes [22].

### 2.1. Sensing Model of SNs and ANs

**Figure 1:**
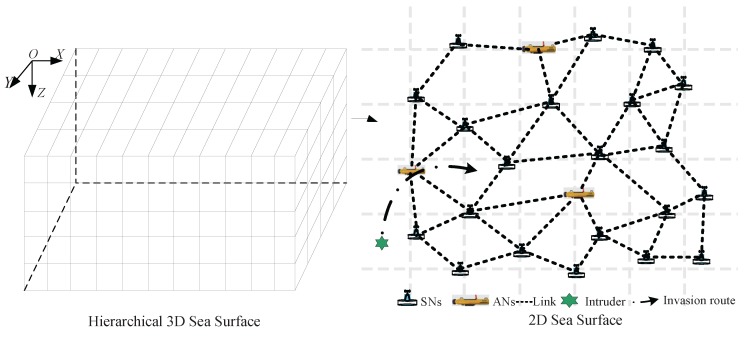
The intrusion detection over actuator-assisted underwater sensor networks.

As shown in the left part of Figure 1, the three-dimensional underwater space is described by small cubes [23]. Each layer of the space can be presented by a two-dimensional surface, as shown in the right part of Figure 1. We assume that each node (i.e., SN or AN) has a Boolean sensing model [24] with the sensing range $R_{s}$, and the communication range is denoted by $R_{c},$ where $R_{c}\geq 2R_{s}$. SNs and ANs can sense the environment and detect intruders within their sensing region, and the sensing region is described by the disk of radius $R_{s}$ centered at the node. In addition, AN can autonomously move in the region of interest (ROI). An intruder is said to be detected by an SN or AN if it has been located inside the sensing region of the SN or AN.

### 2.2. Energy Model for Communication Consumption

In the underwater environment, the energy consumption of nodes in the information transmission process is much greater than the ones in information sensing, processing and receiving [25]. Based on this, we use a common communication consumption model to describe the energy model of SNs and ANs, as proposed in [26]. Define $p_{r}$ as the power threshold for a node to receive the information package, and *d* is the transmitting distance. The energy consumption for transmitting information is denoted by $E_{tx}\left(d\right)$, and it can be calculated as:

(1) $$E_{tx}\left(d\right)=T_{p}\cdot A\left(d\right)\cdot p_{r}$$

where $T_{p}$ denotes the transmitting time of the data package.

In Equation (1), $T_{p}$ is defined as:

(2) $$T_{p}=\frac{M_{b}}{S_{v}}$$

where $M_{b}$ and $S_{v}$ are the size and transmission speed of the information package, respectively.

In addition, A(d) denotes the energy attenuation with the transmitting distance *d*, and it can be calculated as:

(3) $$A\left(d\right)=d^{\lambda }\cdot \beta^{d}$$

where λ is the energy spreading factor related to sensing model (λ is 1 for cylindrical, 1.5 for practical and 2 for spherical spreading).

The parameter $\beta =10^{\alpha (f)/10}$ is determined by the underwater acoustic absorption coefficient α(f), and it can be given as:

(4) $$\alpha \left(f\right)=0.11\frac{10^{-3}f^{2}}{1+f^{2}}+44\frac{10^{-3}f^{2}}{4100+f^{2}}+2.75\times 10^{-7}f^{2}+3\times 10^{-6}$$

where *f* is the frequency of the carrier acoustic signal in kHz, and α(f) is in dB/m.

Denote the time of information package transmitting as $t_{n}$, and the communication range is $R_{t}$. With Equation (1), the energy model for communication consumption in node *i* is denoted by $C_{e}^{i},$ which is shown as:

(5) $$C_{e}^{i}=E_{tx}\left(R_{t}\right)\cdot t_{n}$$

### 2.3. Average Intruder Detection Probability

**Figure 2:**
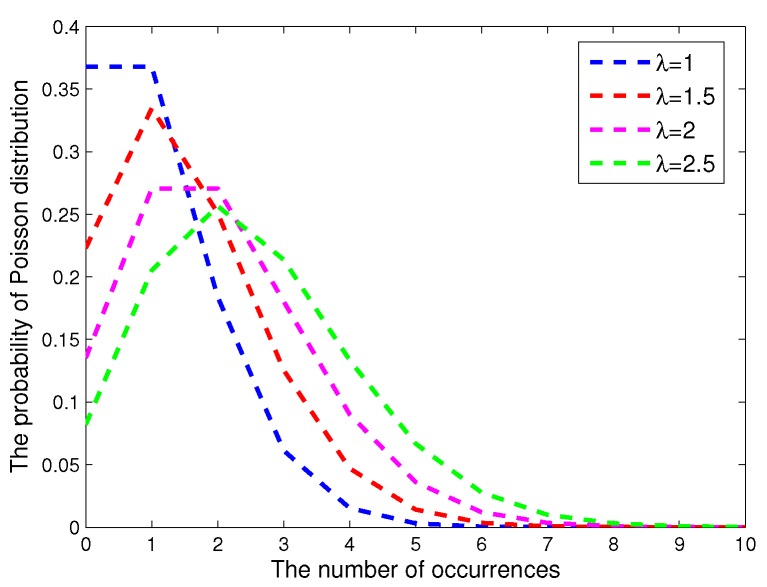
The probability of the Poisson distribution.

Without loss of generality, let the detection area *R* be a thin cuboid, which is partitioned into many virtual lattices. It is assumed that SNs are independently deployed with a random uniform distribution. These SNs can be air-dropped or launched via artillery in battlefields or unfriendly environments. It has been proven in [27] that the Poisson process can well describe the deployment of SNs. Based on this, we denote the density of the Poisson process as λ, and the distribution is described in Figure 2. In a region R, the number of located SNs, i.e., N(R), follows a Poisson distribution with parameter $\lambda ∥R∥$, where $∥R∥$ represents the volume of the region. Then, the probability of the SN quantity is given by:

(6) $$P\left(N\left(R\right)=k\right)=\frac{e^{-\lambda ∥R∥}{\left(\lambda ∥R∥\right)}^{k}}{k!})$$

The coverage rate $P_{c}$ is defined as:

(7) $$P_{c}=1-e^{-\frac{k\pi R_{s}^{2}}{∥R∥}}$$

**Figure 3:**
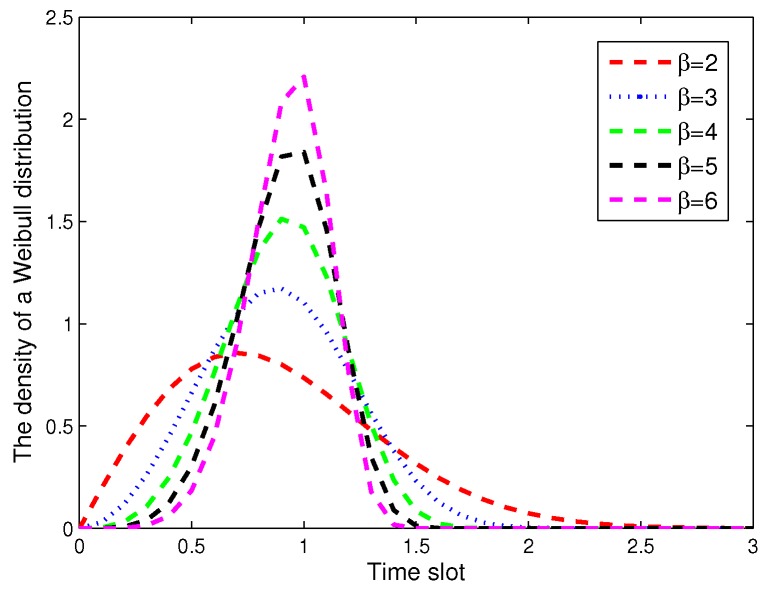
The density of the Weibull distribution.

**Figure 4:**
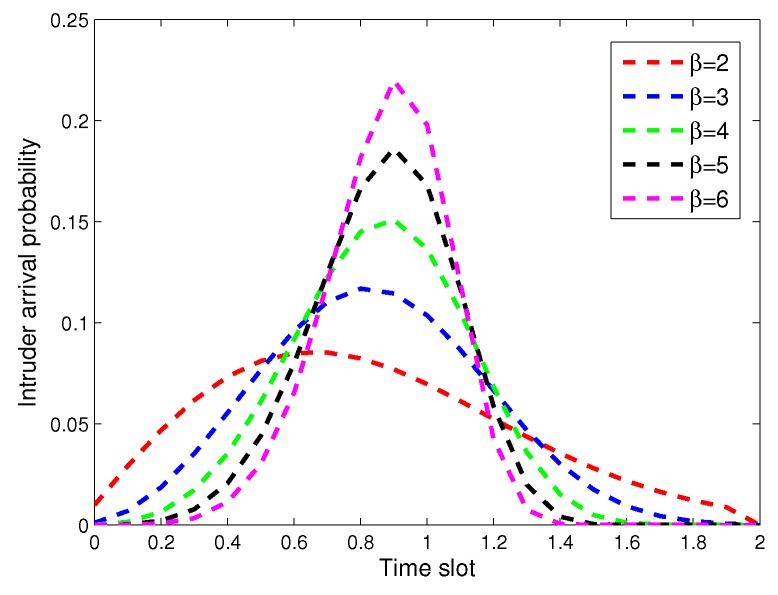
The probability between two arrival times for the intruder.

Obviously, it needs to deploy a large number of static sensors if the coverage rate $P_{c}$ approaches one. In order to improve the coverage rate with a small quantity of SNs, the deployment of ANs is necessary. In general, the intruder is assumed to stochastically arrive at each virtual lattice *i*, where i=1,2,⋯,q. At an arbitrary lattice *i*, the intruder arrival time *t* is a random variable with a distribution of cumulative function F(t). Due to the temporal correlation of intruder arrival times, we use the Weibull distribution to describe the arrival time of the intruder. The Weibull distribution can well characterize the temporal correlation of the arrival time of the intruder, and it has been widely applied to model many real-world random events, especially in a sparse environment. Based on this, the density is denoted as f(t), and Figure 3 is given to show the distribution with different β. The cumulative function F(t) is described in Figure 4, wherein the probability between two arrival times is very small. The definition of F(t) is given as:

(8) $$\begin{array}{cc} f(t) & =\frac{\beta }{\lambda }{\left(\frac{t}{\lambda }\right)}^{\beta -1}e^{-{\left(\frac{t}{\lambda }\right)}^{\beta }} \end{array}$$

(9) $$\begin{array}{cc} F(t) & =1-e^{-{\left(\frac{t}{\lambda }\right)}^{\beta }} \end{array}$$

where t≥0, λ≥0, and β≥1. When β=1, Weibull distribution becomes the well-known Poisson distribution.

**Figure 5:**
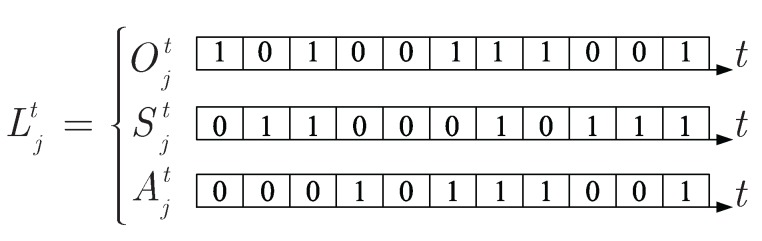
An example of $L_{j}^{t}$ for the the state of lattice *j* at time slot *t*.

**Figure 6:**
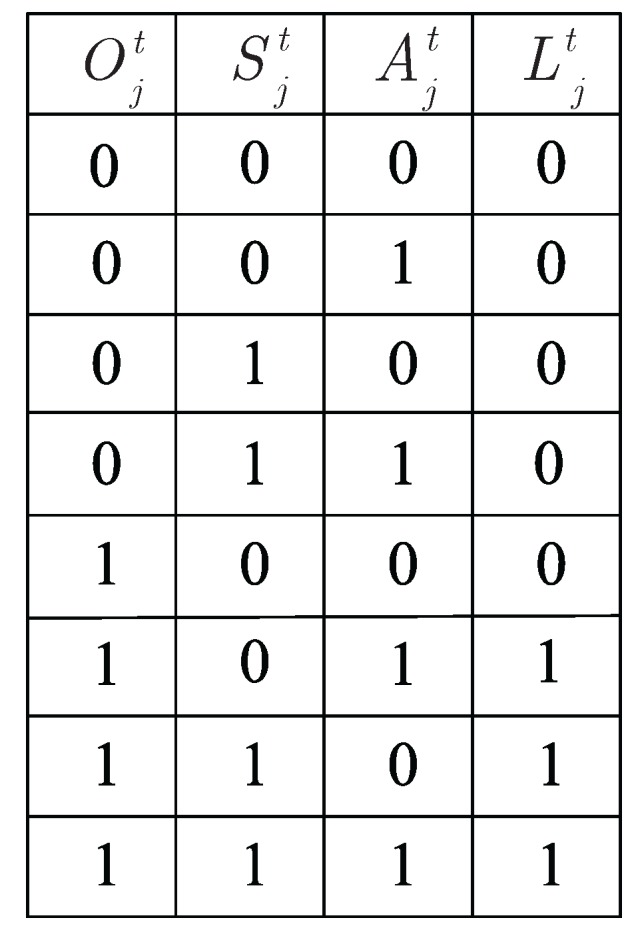
The truth table of state $L_{j}^{t}$.

The state of intruder arrival is denoted by $O_{j}^{t}$. $O_{j}^{t}=1$ if an intruder arrives at lattice *j* during time slot *t*, and $O_{j}^{t}=0$ otherwise. The state of SNs in lattice *j* is denoted by $S_{j}^{t}$. $S_{j}^{t}=1$ if there are at least two SNs during time slot *t* in lattice *j*, and $S_{j}^{t}=0$ otherwise. The state of ANs in lattice *j* is denoted by $A_{j}^{t}$, $A_{j}^{t}=1$ if there is at least one AN staying at lattice *j* at time slot *t*, and $A_{j}^{t}=0$ otherwise. Then, we characterize the state of lattice *j* at time slot *t* as $L_{j}^{t}=(O_{j}^{t}$, $S_{j}^{t}$, $A_{j}^{t})$. Figure 5 illustrates that if an intruder arrives at lattice *j* during time slot *t* and there are at least two SNs, i.e., $O_{j}^{t}=1$ and $S_{j}^{t}=1$, then the intruder is detected. Meanwhile, if an intruder arrives at lattice *j* during time slot *t* and the number of SNs is less than two, however, there is at least one AN staying at lattice *j* during the same time slot, i.e., $O_{j}^{t}=1$, $S_{j}^{t}=0$ and $A_{j}^{t}=1$, then the intruder is also considered to be detected. From the above description, we can draw a truth table of state $L_{j}^{t}$ to show the relationships among $O_{j}^{t}$, $S_{j}^{t}$ and $A_{j}^{t}$. As stated in Figure 6, their relationship can be described as follows:

(10) $$L_{j}^{t}=O_{j}^{t}S_{j}^{t}+O_{j}^{t}{\bar{S}}_{j}^{t}A_{j}^{t}$$

As mentioned above, we define the following important performance metrics.

**Definition 1** (average intruder detection probability): Given a sequence of states $L_{j}^{t}=(O_{j}^{t}$, $S_{j}^{t}$, $A_{j}^{t})$, $j=\left\{1,2,\cdots ,m\right\}$, the average intruder detection probability γ is defined as:

(11) $$\gamma =\lim_{t^{\prime }⟶\infty }\frac{\sum_{t=1}^{t^{\prime }}\sum_{j=1}^{m}O_{j}^{t}S_{j}^{t}+\sum_{t=1}^{t^{\prime }}\sum_{j=1}^{m}O_{j}^{t}{\bar{S}}_{j}^{t}A_{j}^{t}}{\sum_{t=1}^{t^{\prime }}\sum_{j=1}^{m}O_{j}^{t}}$$

In Equation (11), we use states $L_{j}^{t}$ to define the average intruder detection probability γ, which is calculated by the ratio of the number of detected intruders to all arriving intruders. This design models the arrival of the intruder at a specific lattice as a renew process. In Equation (11), the average intruder detection probability γ consists of two parts, i.e., deterministic intruder detection probability θ and monitoring intruder detection probability α. For instance, when the value of γ is 0.5, it means that half of the intruders can be detected by the SNs or ANs. Now, we present the following two definitions.

**Definition 2** (deterministic intruder detection probability): If there is at least one SN in lattice *j*, the intruder can be monitored no matter when it arrives at this lattice. Then, the deterministic intruder detection probability θ is defined as:

(12) $$\theta =\lim_{t^{\prime }⟶\infty }\frac{\sum_{t=1}^{t^{\prime }}\sum_{j=1}^{m}O_{j}^{t}S_{j}^{t}}{\sum_{t=1}^{t^{\prime }}\sum_{j=1}^{m}O_{j}^{t}}$$

**Definition 3** (monitoring intruder detection probability): In the process of AN monitoring, the periodic intruder detection probability α is defined as:

(13) $$\alpha =\lim_{t^{\prime }⟶\infty }\frac{\sum_{t=1}^{t^{\prime }}\sum_{j=1}^{m}O_{j}^{t}{\bar{S}}_{j}^{t}A_{j}^{t}}{\sum_{t=1}^{t^{\prime }}\sum_{j=1}^{m}O_{j}^{t}}$$

### 2.4. Problem Definition

Given the previous discussions, the intrusion detection problem can now be stated as follows.

Problem 1: Considering the energy consumption and topology connectivity constraints, we aim to design a monitoring algorithm for SNs to detect the existence of an attacker in the three-dimensional underwater environment. Based on the lattice topology, we attempt to design a monitor patrolling algorithm for SNs and ANs to optimize the communication links and improve the detection probability.

### 2.5. Graph Preliminaries

In the following, we introduce rigid graph and optimally rigid graph, which are important notions about connectivity in topology optimization scheme. The topology relationship of AN-assisted UWSNs is represented by an undirected graph G. It consists of a center vertex set $V=I_{i}^{〞}\in$$I_{i}=\left\{1,2,\cdots ,m\right\}$, where $I_{i}$ is the ID number of virtual lattices, $h_{i}\in {ℜ}^{3}$ is the positions, $E=\left\{(i,j)\in V\times V,i\;j\right\}$ is the edge set, and (i, j) represents the interconnection edges among the vertices. In addition, the SNs and ANs are denoted by $S_{SN}=\left\{S_{1},S_{2},\cdots ,S_{c}\right\}$ and $S_{AN}=\left\{S_{1},S_{2},\cdots ,S_{n}\right\}$, respectively. This alternating sequence of distinct vertices and edges in the graph is called a ‘path’, and the graph G is ‘connected’ if there is a path between any pair of distinct nodes. Now, we first present the definitions of connectivity and rigid graph.

**Definition 4** (connectivity) [28]: The graph is connected if there is a path between any pair of SNs in the active state.

**Definition 5** (rigid graph) [29]: A framework (or graph) is rigid iff continuous motion of the points of the configuration maintaining the bar constraints comes from a family of motions of all Euclidean space, which are distance-preserving. A graph that is not rigid is said to be flexible.

**Figure 7:**
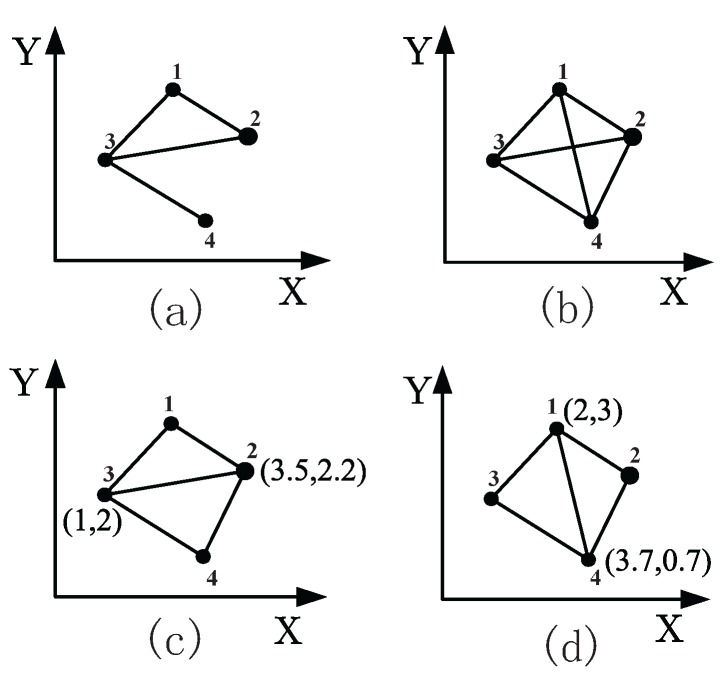
Flexible and rigid frameworks. (**a**) is flexible, while (**b**), (**c**) and (**d**) are rigid. Particularly, (**c**) is minimally rigid, and (**d**) is minimally weighted rigid.

Good connectivity is one characteristic of rigidity. Figure 7a is flexible, because the distance between Vertex 2 and Vertex 4 can be continuously changed freely; while Figure 7b is rigid, in which each vertex has at least two adjacent edges. Another two important notions are minimal rigidity and minimally weighted rigidity. A minimally-rigid graph is a rigid graph that no edge can be removed from without losing rigidity. As shown in Figure 7c and Figure 7d, the two frameworks are the same shape apart from the edge in Figure 7c and the edge in Figure 7d. To compare the two edge weights, the edge is equal to 2.5 less than the edge that equals 3.19. Then, we call the framework in Figure 7c as the minimally-weighted rigidity graph.

Generally, communication consumption of the network comes from two factors: (i) the number of links; and (ii) the sum of the length for all edges. Obviously, these factors are represented by the minimally-rigid graph. Thus, the minimally-rigid graph is adopted in this paper to optimize the communication consumption of the network. In order to ensure the rigid maintenance, the following lemmas are needed.

**Lemma 1** [28]: The initial subgraph formed by one node and its n−1 neighbors is a rigid graph with 2n−3 edges at least if $R_{s}$ satisfies $R_{s}\leq \frac{R_{c}}{\sqrt{8}}$.

Notice that the communication range of a sensor node $R_{c}$ is an arbitrary value; thus, we give Lemma 2 to show the necessary condition to generate a rigid graph in a heterogeneous model.

**Lemma 2** [28]: The initial subgraph formed by one node and its n−1 neighbors is a rigid graph with 2n−3 edges at least if *r* satisfies $r\leq \left(1-DOI\right)R_{m}/\sqrt{8}$.

In framework G, we assume $q_{i}\left(t\right)$ is a differentiable function for each vertex *i*. If V(i, j)∈E satisfies $∥q_{i}-q_{j}∥=\gamma >0$ and ${\left(q_{i}-q_{j}\right)}^{T}\left({\dot{q}}_{i}-{\dot{q}}_{j}\right)=0$ at the initial rotation time t≥0, we say that $\dot{q}=({\dot{q}}_{1}$, ${\dot{q}}_{2}$,⋯, $q_{n})$ is an infinitesimal flex. An infinitesimal flex is trivial if it results from a rigid motion of the framework. A framework is said to be infinitesimally rigid if it only has trivial infinitesimal flex. The infinitesimal rigidity of a framework is a stronger condition than rigidity, where all infinitesimally-rigid networks are rigid. Then, we build a matrix M, whose rows and columns correspond to the edges and coordinates of the vertices, respectively. Then, the row corresponding to the edge (i,j) is given as:

(14) $$\begin{array}{ccccccccccccc} & q_{1}^{1} & q_{1}^{2} & ... & q_{i}^{1} & q_{i}^{2} & ... & q_{j}^{1} & q_{j}^{2} & ... & q_{N}^{1} & q_{N}^{2} & \\ (i,j)[ & 0 & 0 & ... & q_{i}^{1}-q_{j}^{1} & q_{i}^{2}-q_{j}^{2} & ... & q_{j}^{1}-q_{i}^{1} & q_{j}^{2}-q_{j}^{1} & ... & 0 & 0 & ] \end{array}$$

Lemma 3 shows the relationship between the infinitesimally-rigid graph and the rigidity matrix.

**Lemma 3** [28,30]: M is a rigidity matrix of a general structure with *n* vertices. In two-dimensional space, if and only if when rank(M)=2n−3, this structure is infinitesimal rigid.

## 3. Algorithm Description and Analysis

### 3.1. Virtual-Lattice-Based Monitor Algorithm

In this section, we describe the VLM algorithm to detect the arrival of an intruder. The monitoring area is regarded as a thin sea surface in underwater space. Similar to [31], the monitoring area can be divided into multiple equally-sized virtual lattices. Then, a set of SNs is randomly deployed in the sea surface. The roles of SNs are to collect data from the environment and to stay at a lattice to monitor the intruder. In order to better grasp the regional information, a set of ANs is allocated to monitor the intruder. Each AN dynamically changes its position and locally broadcasts a HELLO message with its max transmission range to all nodes at that lattice. Then, we record whether an AN has received the HELLO-ACK message. In the end, SNs and ANs cooperatively monitor the intruder arrival. The detailed process is given in Algorithm 1.

**Algorithm 1 **Virtual Lattice-Based Monitor Algorithm
1:Input: $S_{SN}=\left\{S_{1},S_{2},\cdots ,S_{c}\right\}$, the position of all SNs2:$\;\;\;\;S_{AN}=\left\{S_{1},S_{2},\cdots ,S_{n}\right\}$, the position of all ANs3:With GAF, a cuboid is partitioned into many virtual small lattices, their ID set is denoted by $I=\left\{I_{1},I_{2},\cdots ,I_{m}\right\}$.4:**for** i∈I, (i=1, 2, ⋯, m) **do**5: AN $S_{z}$ locally broadcasts a HELLO message to all nodes in lattice $I_{i}$ with its max transmission range.6: **if** AN $S_{z}$ receives a number of HELLO-ACK message which is greater than or equal to two **then**7:  Record $I_{i}\in P$8: **else**9:  AN $S_{z}$ receives a number of HELLO-ACK message which equal to one10:  Record $I_{i}\in Q$11: **end if**12: Record center coordinates set $I_{i}^{\prime }=(x_{i}$,$y_{i}$,$z_{i})$.13:
**end for**
14:Delete $I_{i}\in P$ and corresponding center coordinates from ID set I and $I_{i}^{\prime }$.15:Record new ID set $I_{i}^{\prime }\in I$, new center coordinates set $I_{i}^{〞}\in I_{i}^{\prime }$.16:Output: The lattices that are required to be patrolled by ANs, and the ID set is $I_{i}^{〞}$.


The objective of Algorithm 1 is to find the lattices that are not satisfied with the two-coverage condition, and these lattices are required to be patrolled by ANs. Inspired by this, each AN locally broadcasts a HELLO message to all nodes in the lattice. By judging the received number of HELLO-ACK, ANs can record the ID set of lattices that are required to be patrolled by ANs. Especially, one-hop transmission is considered in each lattice, because the range of each lattice is relative small. To analyze the performance of Algorithm 1, the following theorem is given.

**Theorem 1**. In VLM, the average intruder detection probability γ is a constant under the ideal situation, where $\gamma =\theta +\frac{k}{m}\cdot \frac{n}{k}=\theta +\frac{n}{m}$, and *n* denotes the number of ANs.

**Proof** **of** **Theorem** **1.** According to Definition 1, the average intruder detection probability γ consists of two independent probability, i.e., the deterministic intruder detection probability θ and the periodic intruder detection probability α. Notice that θ is a deterministic value, and it does not change with time; while, the value of α depends on the number of intruders. In VLM, each AN does not need to know the intruder prior information; however, each lattice can always be selected to be monitored by ANs. It is assumed that the probability of intruder arrival at each time slot is $\bar{F}.$ Then, the number of ANs that have detected intruders is $n\bar{F}.$ In addition, $k\bar{F}$ denotes the remaining lattices that can accommodate the intruders. *k* is the difference between the number of all lattices and the lattices that have detected the intruders. These judgments are based on the $\frac{k}{m},$ which is the premise of the monitoring intruder detection probability. Therefore, γ can be calculated as:

(15) $$\gamma =\theta +\frac{k}{m}\cdot \frac{n\bar{F}}{k\bar{F}}=\theta +\frac{n}{m}$$

which completes the proof. ☐

**Remark 1**: As shown in Algorithm 1, each lattice is always monitored by an SN or AN. Based on this, the intruder can be detected by the AN-assisted UWSNs. If θ, *n* and *m* do not change with time, the average intruder detection probability γ will be a fixed value.

### 3.2. The Optimal and Coordinate Lattice Monitor Algorithm

In order to improve γ, this section proposes the OCLMP algorithm, which can be regarded as a weak barrier coverage. This algorithm is divided into two steps, and the first step is to generate and optimize the network topology. The optimized topology has good connection and low energy dissipation. If an intruder appears at a lattice with a optimized topology, the intruder can be detected. The second step is to coordinate the monitor patrolling of ANs. With the collaboration of ANs, the AN-assisted UWSNs can yield a much higher average intruder detection probability γ compared with the result in Section 3.1.

The OCLMP algorithm is described in Algorithm 2. A rigid graph is generated according to its neighbor rules among SNs (see Lines 1–9 in Algorithm 2). To decrease the complexity of the initial rigid topology, we delete some links that do not belong to global optimally-rigid graph by using locally-collected information (see Lines 1–17). Based on this, a low-energy dissipation network that is at least two-connected can be built.

**Algorithm 2 **Optimal Lattice Monitor Path Algorithm
1:Input: $s_{j}\in S\in S_{c}$2:Compute all the neighbor links $E_{ij}$ and sequence it by length in an increasing way3:Initialize the $M_{i}$, let $M_{i}=M_{i}^{\Delta }\left(1\right)$4:
$j=1:\left|E_{ij}\right|$
5:
**if** $\left(rank\left(M_{i}\right)\leq 2\left(\left|I_{i}^{〞}\right|+1\right)-3\right)$ **then**
6:
$\;\;\;\;M_{i}=\left[\begin{array}{c} M_{i}\\ M_{i}^{\Delta }\left(j+1\right) \end{array}\right]$
7: **if** $(M_{i}$ is full rank) **then**8:  According to $M_{i}$, record the edge $E_{ij}^{\prime }$9: **end if**10:
**end if**
11:**for** $l=\{I_{i}^{〞}$, i}**do**12: **for** $k=\{I_{i}^{〞}$, *i*, k≠l} **do**13:  **if** ((k,$l)\notin E_{ij}^{\prime })$ **then**14:   Delete (k,l)15:   Draw the edge of $E_{ij}^{\prime }$16:  **end if**17: **end for**18:
**end for**



As shown in Algorithm 2, the optimized graph can be given through the “drawing” and “deleting” implementations. In a broadcast medium, the implementations of “drawing” and “deleting” mean the changes of topology. That is to say, we can change the communication topology of UWSNs to design a desired routing relationship of SNs and ANs. If they are in each other’s transmission range, the deletion implementation means the change of topology. Similarly, the implementation of adding is also the change of topology. If intruders break into a lattice from any direction, they can be detected by the AN-assisted UWSNs. Next, a theorem is first introduced.

**Theorem 2**. In OCLMP, $\gamma \geq \frac{n+s}{m}$, where *m* is the total number of monitoring lattices.

**Proof** **of** **Theorem** **2.** This proof is similar to the proof in Theorem 1. Denote the average probability of intruder arrival at each time slot by $\bar{F}$. Hence, the number of all lattices that can monitor intruders is $m\bar{F}$. All actuators can detect the number of intruders $n\bar{F}$; $s\bar{F}$ is the number of lattices that can monitor intruders doubtlessly. Then, pulsing $n\bar{F}$ with $s\bar{F}$, this means the total number ANs and SNs can detect the intruders at time slot *t*. The average intruder detection probability γ can be calculated as:

(16) $$\gamma =\frac{n\bar{F}+s\bar{F}}{m\bar{F}}=\frac{n+s}{m}$$

which completes the proof. ☐

**Remark 2**: In Theorem 2, when *t* goes to infinity, γ remains at the same value. However, the case of the value of γ equaling $\frac{n+s}{m}$ is an ideal situation. In general, the locations of SNs do not follow a uniform distribution. Inspired by this, we deploy ANs to patrol and collaborate with SNs. As stated in Lemma 3, the geometric relationships between neighbor nodes can expand the monitoring scope. Thus, the intruders can be detected if they travel through the expanded monitoring scope. Hence, it can be obtained that $\gamma >\frac{n\bar{F}+s\bar{F}}{m\bar{F}}$.

In order to find the shortest patrolling path, an equal price search strategy is further given for ANs. From Algorithm 2, some independent lattices are not monitored by ANs or SNs. These independent lattices are put in a set Q, and $I_{i}\in Q$ is its ID number, where i=1, 2 , ⋯, m. With the equal price search method, all of the independent lattices can be linked to the shortest road by the ID number. That is to say, this method is to find the shortest road for AN monitor patrolling. There are five points, i.e., A, B, C, D and E. Begin with Point A, and expand it by the ascending order to four new nodes, AB (7), AC (5), AD (3) and AE (10), where 7, 5, 3 and 10 are the distances between AB, AC, AD and AE, respectively. In addition, mark A as an expanded node, while AB, AC, AD and AE as waitingexpanded nodes. Compare the distance information among the four new nodes, and find the shortest distance to expand continuously. Then, AD (3) has the shortest distance, and we expand it to three new nodes, i.e, ADB (6), ADC (9) and ADE (11). Meanwhile, mark AD as the expanded node, and ADB, ADC and ADE as waiting expanded nodes. Then, comparing all of the waiting expand nodes, AC (5) has the shortest distance. Once more, expand AC to three new nodes ACB (8), ACD (13), ACE (14), and mark AC as the expanded node, while ACB, ACD, ACE are marked as the waiting expanded nodes. Update the waiting expand nodes, and find the node that has the shortest distance. ADB has the shortest distance. Then, repeat the above operation until all five points appear in a new expanded node. The shortest link among A, B, C, D and E can be built up. At last, ANs are assigned to monitor these independent lattices along this link. The pseudocodes can be found in Algorithm 3.

**Algorithm 3 **Equal Price-Basedd Search Method With Coordination SNs Monitor Patrolling Algorithm
1:Input: New ID set $I_{i}\in Q$, (i=1, 2, ⋯, m) and sequence it in an increasing way.2:Initialization, set the min ID $I_{i}$ in the waiting expand node list $D_{k}$, set the have expanded node list $H_{k}=0$.3:Take $I_{i}$ from $D_{k}$ and expand $I_{i}$ to $I_{i}I_{i+1}$, $I_{i}I_{i+2}$, ⋯, $I_{i}I_{m}$, meanwhile record the corresponding distance $d_{i,i+1}$, $d_{i,i+2}$, ⋯, $d_{i,m}$. At last, put $I_{i}I_{i+1}\left(d_{i,i+1}\right)$, $I_{i}I_{i+2}\left(d_{i,i+2}\right)$, ⋯, $I_{i}I_{m}\left(d_{i,m}\right)$ into $D_{k}$ and $I_{i}$ into $H_{k}$, respectively.4:Take $\min D_{k}$ continue to expand, i.e., $I_{i}I_{i+2}\left(d_{i,i+2}\right)$ is the minimum node, expand it to $I_{i}I_{i+2}\left(d_{i,i+2}\right)$, $\left(j∼i,j∼i+2\right)$, then update $D_{k}$ and $H_{k}$.5:Continue Step 4 until a new expanded lattice with all of the ID sequence $I_{i}I_{i+1}I_{i+2}I_{m}$ appeared.6:Set t=0, $I_{i}=0$. According to the new ID sequence, assign each ANs to monitor one lattice.7:If there is no ANs monitoring at ID $I_{i}$, Set $I_{i}=I_{i}+1$; otherwise $I_{i}=0$8:Call algorithm 2.9:t=t+1, If an AN detects an intruder at time slot t−1, then it claims itself not busy. Count the total number of not busy ANs $\bar{n}$, if $\bar{n}>0$, continue.10:The $\bar{n}$ ANs are newly selected to monitor lattices with ID $I_{i}=I_{i}+1$.11:Continue Step 6 until all ANs run out of energy.


## 4. Simulation Results

In this section, simulation results are provided to validate the effectiveness of the proposed method. A stationary two-dimensional Poisson point process is used to model the locations of SNs. Meanwhile, each virtual lattice is considered to be fully covered if at least one SN is located in this lattice. Accordingly to Lemma 3, the maximal transmission range is chosen as $R_{m}=\sqrt{8}/\left(1-DOI\right)$, where DOI=0.1. The length of each virtual lattice is designed as a constant, i.e., r=5. Similar to the assumption in [32], the arrival time of the intruder in this paper is assumed to follow the independent and identically distributed Weibull distribution. In addition, it is designed that λ=10.

**Figure 8:**
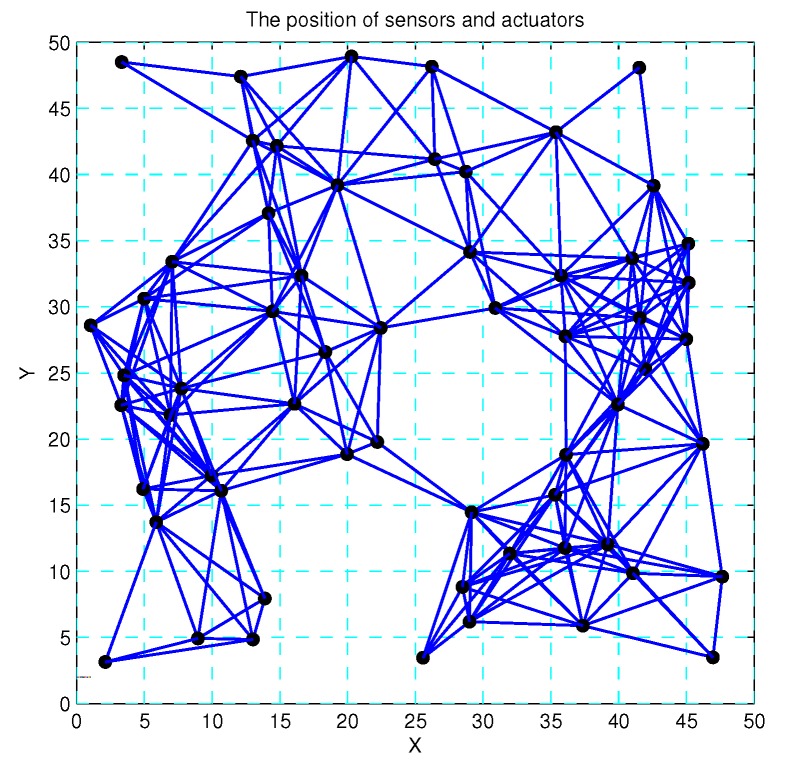
60 sensors randomly deployed in 50×50 region.

**Figure 9:**
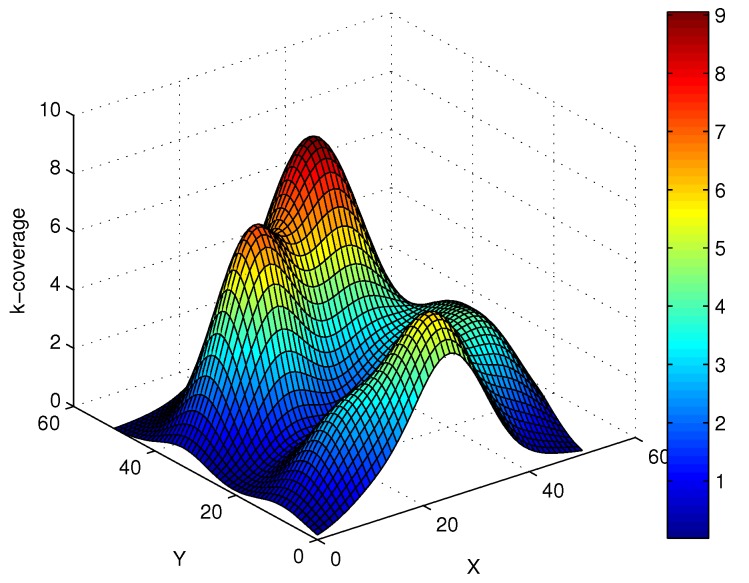
The k-coverage of the region by the sensors.

**Figure 10:**
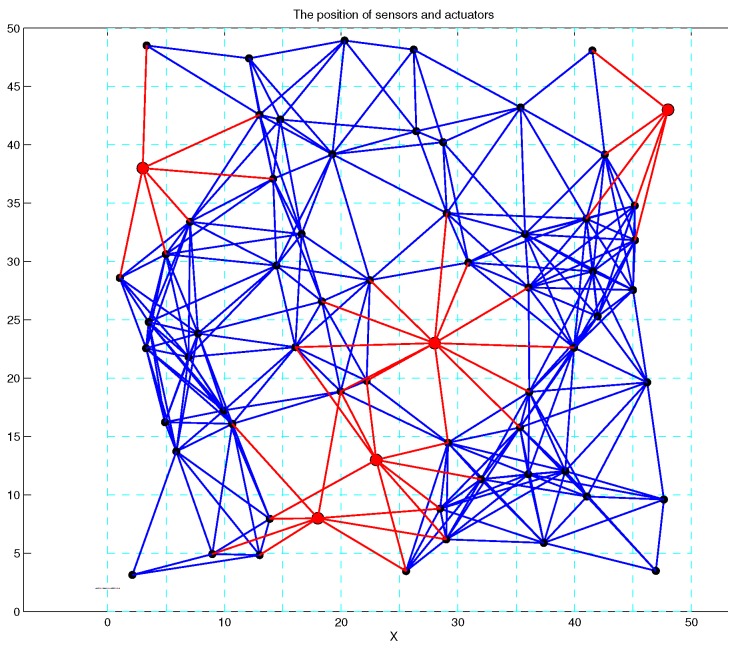
The wireless sensors and actuators’ network.

**Figure 11:**
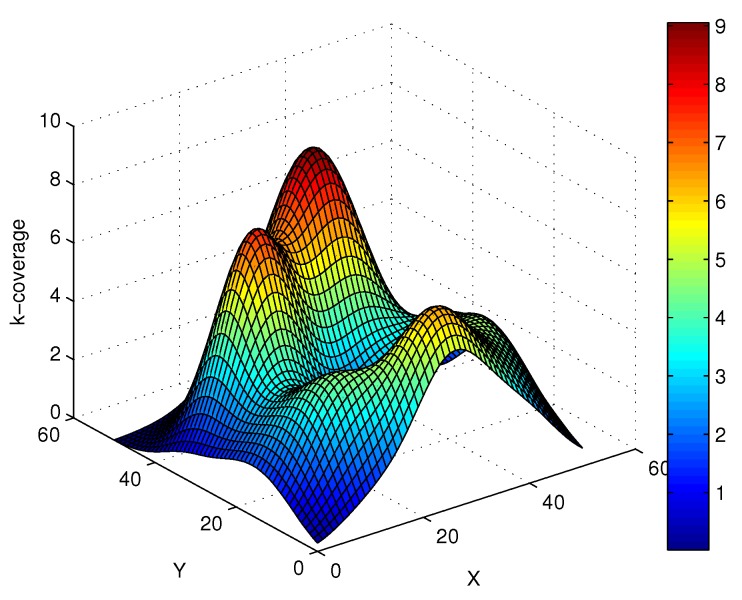
The improved coverage via activated actuators.

Based on the above design, 60 SNs are randomly deployed in the 50×50 region, and this region is divided into 100 virtual lattices. In Figure 8, the positions of SNs follow a Poisson distribution, and there exist gaping holes in the coverage lattice. Meanwhile, Figure 9 shows the coverage results of SNs, and it can been seen that most of the region can achieve multiple coverage. In order to improve the coverage rate and intrusion detection probability, the ANs in corresponding lattices are activated, and it can be seen that the region is fully covered by the SNs and ANs as shown in Figure 10. Obviously, the AN-assisted UWSNs can achieve full coverage with a minimal amount of activated ANs. Correspondingly, the invasive monitoring probability can be improved, and the improved full coverage can be seen in Figure 11.

**Figure 12:**
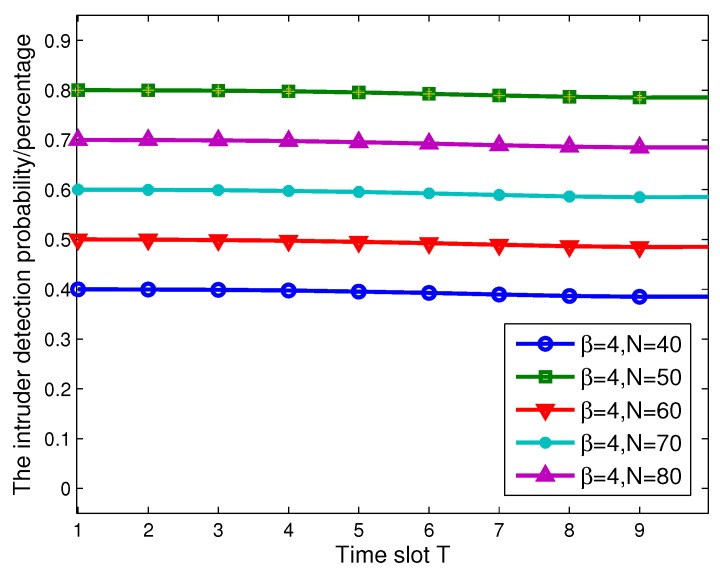
The intruder detection probability γ varies with *T* and *N*, which contains actuator nodes (ANs) and sensor nodes (SNs).

Next, we evaluate the performance of BLMP, which is performed in an ideal condition. In Figure 12, we fix β=4, and the number of ANs is n=10. When N=40, which includes 30 SNs, the average intruder detection probability γ does not change with time slot T, as shown in Figure 12. To be more convincing, we change the value of *N* to 50, 60, 70 and 80. From Figure 12, it can be obtained that γ does not change during the continuous monitoring time slot T, and the values of γ satisfy the result in Theorem 1.

**Figure 13:**
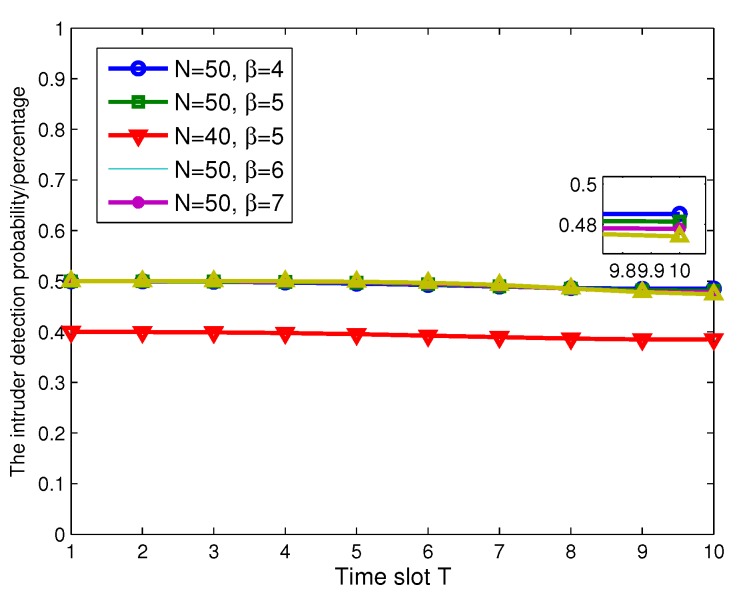
The intruder detection probability γ varies with *T* and β.

**Figure 14:**
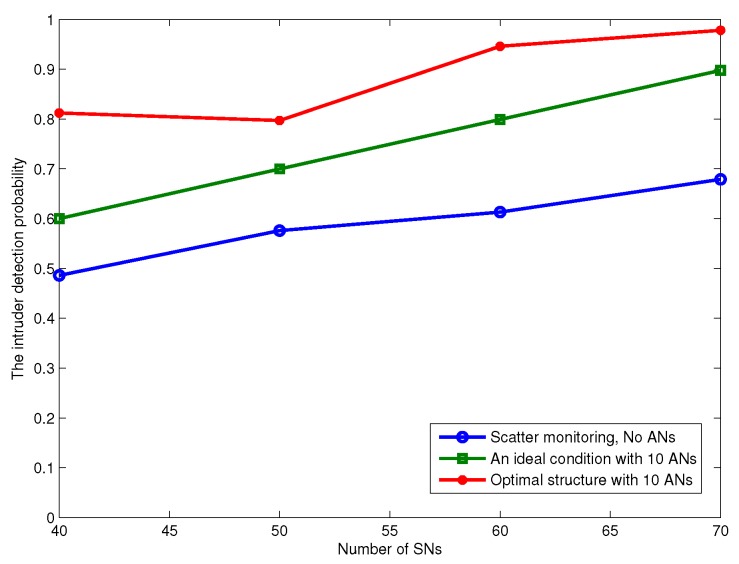
The intruder detection probability γ varies with different SNs’ number in different conditions.

**Figure 15:**
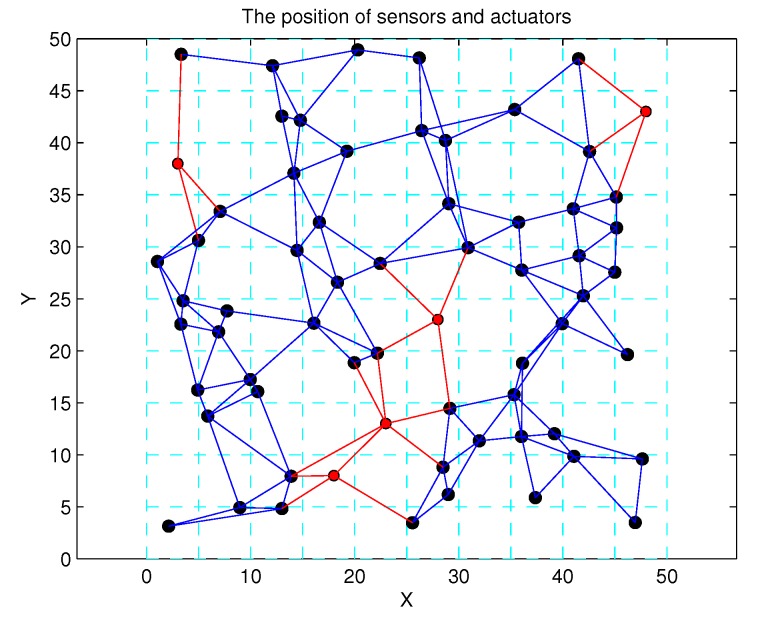
The optimal network structure in Algorithm 2.

**Figure 16:**
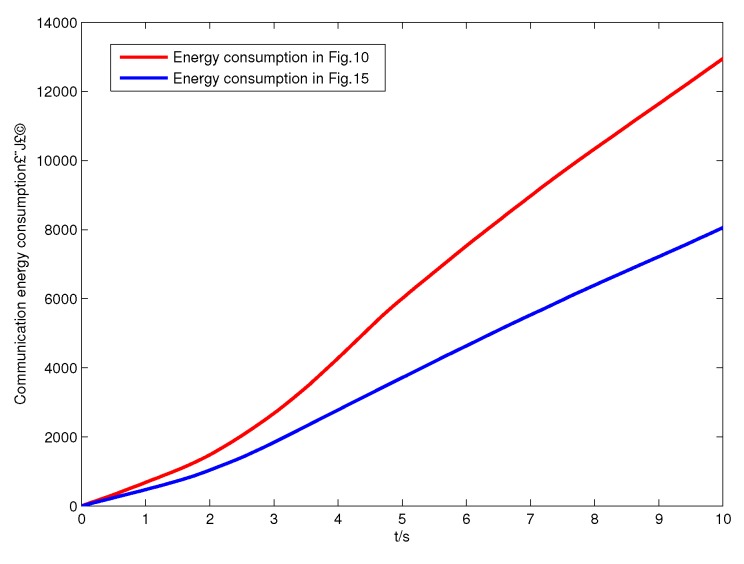
The energy consumption comparison between the two network topologies.

To investigate the impact of β on γ, we give a similar simulation result in Figure 13. Keeping N=50 (40 SNs and 10 ANs), we fix the values of β to 4, 5, 6 and 7, and the results are the same when time slot *T* is varying. Finally, we set N=40 and β=5 as a comparison in Figure 14. It is clearly shown that only *N* can impact the value of γ. Intuitively, the optimal network structure constructed in Algorithm 2 is shown in Figure 15. According to (5), the energy consumption of SNs is computed, and Figure 16 shows the energy consumption comparison between the two topology structures. Compared with the non-optimization topology [20], the optimized network structure in this paper can improve the energy efficiency.

## 5. Conclusion and Future Work

In this paper, we investigate the underwater intrusion detection problem with AN-assisted UWSNs. In order to detect the attacker, a virtual-lattice-based monitor patrolling algorithm is first proposed. Meanwhile, an optimal and coordinative lattice-based monitor patrolling algorithm is also provided to reduce the redundancy of communication links and improve detection probability. In addition, an equal price search strategy is further given for ANs to find the shortest patrolling path. Different from the previous works, the optimized topology can save the communication energy consumption, and the mobility of ANs can improve the detection probability. In the end, simulation results are provided to show the effectiveness of the proposed method.

In our future work, the cooperation control of multiple ANs can be designed to further expedite the underwater intrusion detection problem, such that the ubiquitous monitoring capability can be enhanced with the cooperation of SNs and ANs.
